# Lack of efficacy of Doxil® in TNF-*α*-based isolated limb perfusion in sarcoma-bearing rats

**DOI:** 10.1038/sj.bjc.6601688

**Published:** 2004-04-27

**Authors:** T L M ten Hagen, S Hoving, G Ambagtsheer, S T van Tiel, A M M Eggermont

**Affiliations:** 1 Laboratory of Experimental Surgical Oncology, Department of Surgical Oncology, Erasmus MC-Daniel den Hoed Cancer Center, PO Box 1738, Rotterdam 3000 DR, The Netherlands

**Keywords:** Doxil®, tumour necrosis factor-alpha, isolated limb perfusion, rat, sarcoma

## Abstract

Here we show that Doxil® has minimal antitumour activity in the isolated limb perfusion (ILP) setting and its activity was not enhanced by the addition of tumour necrosis factor (TNF). Doxil® accumulation in tumour tissue was low and also not augmented by TNF. In contrast, activity of free conventional doxorubicin was enhanced by TNF. We conclude that application of Doxil® in a TNF-based ILP is not a useful alternative to free conventional doxorubicin or melphalan.

Isolated limb perfusion (ILP) provides an excellent tool in the treatment of locally advanced tumours. During ILP, high local drug concentrations are possible due to minimal leakage into the systemic circulation, and the effect on vital organs is limited, allowing high dosages to be used. We and others have demonstrated that addition of tumour necrosis factor (TNF) to an ILP with melphalan increased the tumour response dramatically as compared to melphalan alone (Lienard *et al*, 1992; Eggermont *et al*, 1996).

Melphalan is used most commonly in ILP, but other agents have been applied with varying success in limb or organ perfusion (Rossi *et al*, 1992; Weksler *et al*, 1994; Abolhoda *et al*, 1997). We observed in ILP that local toxicity was dose-limiting at suboptimal doxorubicin concentrations (Van Der Veen *et al*, 2000).

The formulation of doxorubicin in long-circulating liposomes (Stealth liposomal doxorubicin, Doxil®) prolongs circulation time, decreases toxicity and augments localisation in tumour tissue (Gabizon *et al*, 2003). We hypothesised that the use of Doxil® in ILP may reduce local toxicity while augmenting tumour accumulation and improving tumour response. In this study, we examined the efficacy of Doxil® in a TNF-based ILP in sarcoma-bearing rats.

## MATERIALS AND METHODS

### Chemicals

Human recombinant TNF-*α* was kindly provided by Dr G Adolf (Bender Wien GmbH, Wien, Austria). Pegylated liposomal doxorubicin (Doxil®, Caelyx™) was kindly provided by Dr Working (ALZA Corporation, Mountain View, CA, USA). Doxorubicin hydrochloride (adriblastina) was purchased from Pharmacia (Brussels, Belgium).

### Animals and tumour model

Male inbred BN rats (soft-tissue sarcoma model, BN175) and WAG/RIJ rats (osteosarcoma model, ROS-1) were obtained from Harlan-CPB (Austerlitz, the Netherlands). Small fragments (3 mm) of tumour were implanted subcutaneously in the right hindleg, as previously described (de Wilt *et al*, 1999). Tumour growth was recorded by calliper measurements, and tumour volume was calculated using the formula 0.4(*A*^2^*B*) (where *B* represents the largest diameter and *A* the diameter perpendicular to *B*). All animal studies were done in accordance with protocols approved by the Animal Care Committee of the Erasmus University Rotterdam, the Netherlands (Workman *et al*, 1998).

A tumour response indicates either a partial remission (PR, decrease of tumour volume between –25 and 90%) or a complete remission (CR, tumour volume less than 10% of initial volume).

### Isolated limb perfusion protocol

Rat limbs were perfused as previously described (de Wilt *et al*, 1999). Tumour necrosis factor (50 *μ*g), Doxil® or doxorubicin (400 *μ*g BN175 and 200 *μ*g ROS-1) were added as boluses to the oxygenation reservoir. Control rats were perfused with Haemaccel or placebo liposomes alone. The concentration of TNF was adapted from previous animal studies, and doxorubicin concentrations that yielded no local toxicity were used. All animal studies were approved as stated above (Workman *et al*, 1998).

### Assessment of doxorubicin accumulation in solid tumour during ILP

Accumulation of doxorubicin in tumour and muscle was determined directly after ILP, as previously described (Mayer *et al*, 1989; Van Der Veen *et al*, 2000). As the ILP included a thorough washout, there was no intravascular doxorubicin present. All animal studies were approved as stated above (Workman *et al*, 1998).

### Statistical analysis

The results were evaluated for statistical significance using the Mann–Whitney *U*-test with SPSS for Windows. *P*-values below 0.05 were considered statistically significant.

## RESULTS

### 

#### Tumour response to Doxil® in TNF-based ILP

**Figure 1:**
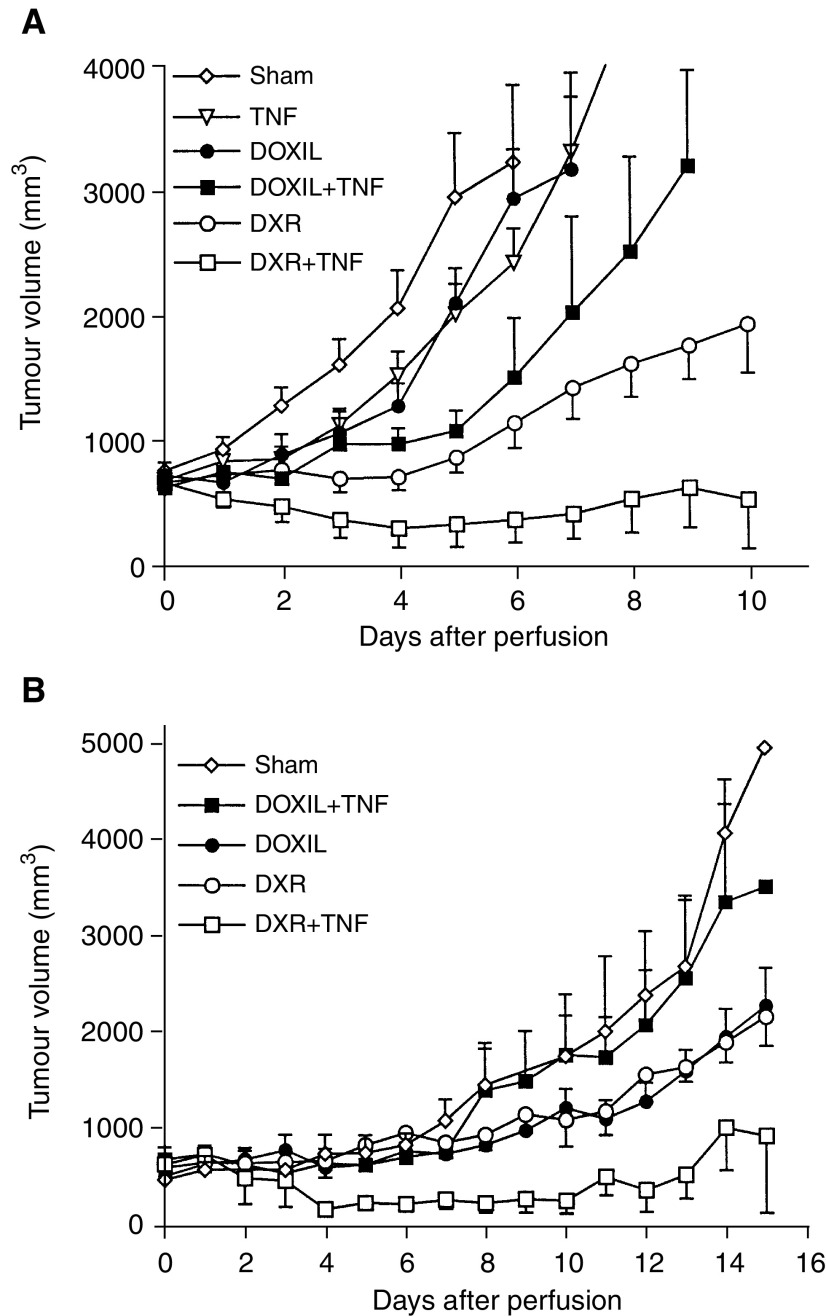
(**A**) Tumour volumes of subcutaneous implanted soft-tissue sarcoma BN175 after isolated limb perfusion with perfusate alone (*n*=6), 400 *μ*g Doxil® (*n*=4), 50 *μ*g TNF (*n*=6), Doxil® plus 50 *μ*g TNF (*n*=8), 400 *μ*g free doxorubicin (DXR) (*n*=7), or a combination of TNF and free DXR (*n*=6). (**B**) Tumour volumes of subcutaneous implanted osteosarcoma ROS-1 after isolated limb perfusion with perfusate alone (*n*=6), 200 *μ*g Doxil® (*n*=6), 50 *μ*g TNF (*n*=8), 200 *μ*g Doxil® plus 50 *μ*g TNF (*n*=7), 200 *μ*g free doxorubicin (DXR) (*n*=6), or a combination of TNF and DXR (*n*=6). The mean tumour volumes are shown±s.e.

Perfusion with Doxil®, TNF or buffer alone resulted in progressive disease in all soft-tissue sarcoma-bearing rats (Figure 1A

). Perfusion with Doxil® plus TNF resulted in a short growth delay followed by rapid outgrowth of the tumour, and all rats showed progressive disease. Application of free conventional doxorubicin resulted only in a slight inhibition of the tumour growth, and no rats showed a tumour response. Isolated limb perfusion with conventional doxorubicin combined with 50 *μ*g TNF increased the antitumour activity with a response rate of 83% (PR and CR combined) (*P*<0.01 compared with doxorubicin alone).

Isolated limb perfusion in osteosarcoma-bearing rats with buffer or conventional doxorubicin alone had no significant effect on tumour growth (Figure 1B). Isolated limb perfusion with TNF alone resulted in a response rate of 25%. Isolated limb perfusion with conventional doxorubicin combined with TNF further increased the tumour response to 83% (*P*<0.05 compared with TNF alone or doxorubicin alone). Isolated limb perfusion with Doxil® only, induced slight tumour growth delay comparable to free conventional doxorubicin. Strikingly, ILP with Doxil® plus TNF diminished the tumour response, and none of the rats showed a tumour response.

### Accumulation of doxorubicin in solid tumour after ILP

We observed that addition of TNF did not significantly augment the accumulation of Doxil® in soft-tissue sarcoma or osteosarcoma when compared to ILP with Doxil® alone (data not shown). Levels of doxorubicin were significantly increased when TNF was added to ILP with free doxorubicin (Van Der Veen *et al*, 2000).

### DISCUSSION

In the present study, we demonstrated that ILP treatment with Doxil® combined with TNF in sarcoma-bearing rats does not provide a useful alternative to free conventional doxorubicin. The lack of efficacy of Doxil® is not due to failure of the drug to be active at the tumour site, as dramatic synergy between Doxil® and TNF after systemic treatment has been shown (ten Hagen *et al*, 2000). Rather, we speculate that the liposomes are unable to extravasate into the tumours during the relatively short ILP interval.

In spite of the indicated usefulness of doxorubicin in ILP for the treatment of sarcoma, we and others observed dose-limiting local toxicity after a TNF-based ILP with conventional doxorubicin (Di Filippo *et al*, 1999; Van Der Veen *et al*, 2000). Biodistribution and pharmacokinetic studies with Doxil® demonstrated a favourable profile of the liposomal formulations over the free drugs, that is, circulation time was extended, toxicity reduced and tumour localisation was increased (Gabizon *et al*, 2003). Therefore, we envisioned that Doxil® could be a good alternative to free conventional doxorubicin in ILP. However, Doxil® failed to induce any response in sarcoma-bearing rats, even when applied in combination with TNF. Minimal accumulation of Doxil® in tumour after ILP with or without TNF was observed, whereas considerably higher levels of doxorubicin were found in tumour after ILP with free doxorubicin plus TNF. A possible explanation for the difference in accumulation between free conventional doxorubicin and liposomal doxorubicin is the particle size. Whereas distribution of a small molecule like doxorubicin is diffusion dependent, the transport of particulate matter is convection dependent (Jain *et al*, 1990; Hobbs *et al*, 1998). This would indicate that, during ILP, drug distribution is mostly diffusion dependent and not convection dependent.

The increased tumour localisation of Doxil seen after systemic administration is reportedly due to the ability of the pegylated liposomes to avoid accumulation in the liver and spleen and other parts of the mononuclear phagocytic system (MPS), which results in a long circulation time and extravasation through the leaky vasculature of tumours (Gabizon *et al*, 2003). The volume of distribution of Doxil® is markedly smaller than that of doxorubicin given systemically, reflecting the broad tissue distribution of the latter. The short 30-min circulation time imposed by the ILP procedure is likely inadequate for the circulation time advantage of Doxil® to have an effect on its distribution, and, of course, the restriction of circulation to the isolated limb obviates the value of avoiding the MPS. Thus, the rapid distribution properties of the small doxorubicin molecule makes it a better choice for ILP procedures, particularly when it is used in combination with TNF.
